# Prevalence and genotypes of *Chlamydia psittaci* in birds and related workers in three cities of China

**DOI:** 10.1371/journal.pone.0308532

**Published:** 2024-08-08

**Authors:** Ling Hou, Jing Jia, Xincheng Qin, Ming Fang, Shengnan Liang, Jianping Deng, Bei Pan, Xiangyuan Zhang, Bin Wang, Conglin Mao, Lihong Cheng, Jie Zhang, Chunhui Wang, Xuewei Ming, Tian Qin

**Affiliations:** 1 Department of Epidemiology, School of Public Health, Shanxi Medical University, Taiyuan, China; 2 National Key Laboratory of Intelligent Tracking and Forecasting for Infectious Diseases, National Institute for Communicable Disease Control and Prevention, Chinese Center for Disease Control and Prevention, Beijing, China; 3 Qingdao Municipal Centre of Disease Control and Prevention, Qingdao, Shandong, China; 4 Shandong Center for Disease Control and Prevention, Jinan, Shandon, China; 5 Liaocheng Center for Disease Control and Prevention, Liaocheng, Shandong, China; 6 Zigong Center for Disease Control and Prevention, Zigong, Sichuan, China; 7 Jiaozhou City Center for Disease Control and Prevention, Qingdao, Shandong, China; 8 Shibei District Center for Disease Control and Prevention, Qingdao, Shandong, China; Huadong Research Institute for Medicine and Biotechniques, CHINA

## Abstract

*Chlamydia psittaci*—a zoonotic pathogen in birds—may be transmitted to humans, causing severe respiratory disease. Individuals working in or living near poultry farms are highly susceptible to *C*. *psittaci* infection. In this study, we assessed the prevalence and genotypes of *C*. *psittaci* in poultries and humans in three cities of China by collecting fecal samples from different poultry species and throat swab samples and serum samples from workers in poultry farms and zoos. These samples were screened by real-time fluorescence quantitative PCR (qPCR) targeting *C*. *psittaci ompA*. The positive samples were subjected to PCR amplification and sequencing of *ompA*. The strains detected in the samples were genotyped on the basis of the phylogenetic analysis of *ompA* sequences. In total, 3.13% (40/1278) poultry fecal samples were positive in the qPCR assay, whereas 3.82% (6/157) of throat swab samples and 42.59% (46/108) of serum samples from the workers were positive in the qPCR and indirect fluorescent antibody assays, respectively. The strains detected in the 32 poultry samples and 6 human samples were genotyped as type A, indicating that the workers were infected with *C*. *psittaci* that originated in poultry birds in farms. Additionally, eight peacocks showed strains with the genotype CPX0308, which was identified in China for the first time. Elucidating the distribution of *C*. *psittaci* in animals and poultry-related workers may provide valuable insights for reducing the risk of *C*. *psittaci* infection within a population.

## Introduction

The genus *Chlamydia* comprises cell-specific parasitic pathogens with a wide distribution that cause infections in various animals and humans [1]. The Chlamydia family has one *Chlamydia* genus, which includes 14 validly published species and four *Candidatus* species. With the expansion of human activities, the discovery of new *Chlamydia* species is gradually increasing [2, 3]. Of these *C*. *psittaci* is a key zoonotic pathogen.

*C*. *psittaci* causes avian chlamydiosis in birds; it has been reported that *C*. *psittaci* can infect up to 30 orders and 467 species of birds and several mammals and humans [4]. The severity of symptoms in birds infected with *C*. *psittaci* varies, and the infection manifests in acute, subacute, or chronic forms or result in asymptomatic carriers [5]. These asymptomatic birds carry the pathogen for a long time and release it into the environment under stress conditions [6].

Transient exposure to *C*. *psittaci*—mainly through the inhalation of aerosols from feathers or feces of birds carrying the pathogen—may cause infection in humans, leading to psittacosis [7]. In general, pet shop owners, zoo administrators, veterinarians, slaughter house workers, foreign and domestic bird keepers, and pet bird enthusiasts are high-risk groups for contracting this pathogen [7, 8]. The severity of symptoms of psittacosis in infected individuals varies, ranging from asymptomatic and mild flulike symptoms to severe acute clinical symptoms [9].

Currently, *C*. *psittaci* is classified into 15 genotypes on the basis of their *ompA* gene sequences, including nine common genotypes (A–F, E/B, M56, and WC) and six temporary genotypes (1V, 6N, MatI16, R54, YP84, and CPX0308) with different levels of host specificity [10]. Typically, the A and B genotypes infect birds of the Psittaciformes and Columbiformes, respectively; the C genotype is typically isolated from ducks and geese; the D genotype is found in turkeys; and the F genotype is also found in turkeys and birds of the family Arrowhead [11]. The E genotype has multiple hosts and has been isolated from pigeons, ducks, and turkeys. Finally, the E/B, WC, and M56 genotypes have been detected in ducks, cows, and rats, respectively [7].

It has been reported that the rise in the number of domestic pet birds and the in-creased contact between wild and captive birds has increased the risk of transmission of *C*. *psittaci* and the number of human infections [12]. Although *C*. *psittaci* infection has been reported worldwide, most studies have focused on patients and birds showing clinical signs of disease. However, to our knowledge, the prevalence of *C*. *psittaci* infection in birds and poultries and workers in different types of facilities in China has few reports [13, 14]. Understanding the prevalence of *C*. *psittaci* in animals and the environment will help to reduce the risk of transmission and outbreaks. The present study investigated the prevalence of *C*. *psittaci* infection in birds and poultries and workers in farms and zoos. Fecal samples from birds and poultries and throat swab samples from employees of farms and zoos in three cities in China were screened using real-time fluorescence quantitative PCR (qPCR) targeting the *C*. *psittaci ompA* gene. In addition, the *C*. *psittaci* strains in the qPCR-positive samples were genotyped to identify the prevalent strains in these regions.

## Materials and methods

### Ethics statement

The study was approved by the ethics committee of the Chinese Center for Disease Control and Prevention and National Institute for Communicable Disease Control and Prevention (ICDC-202115). Written informed consents were obtained from the study participants.

### Sample collection

The sampling process occurred at four distinct intervals: on June 23–28 and September 27–30 in 2022, as well as on January 9–11 and May 9 in 2023. A total of 1278 avian fecal samples (Table 1) were obtained from diverse establishments located in three Chinese cities, including commercial farms, backyard farms, zoos, and slaughterhouses (Table 1). The avian samples encompassed 11 different avian species. The avian and poultry fecal samples collected in Liaocheng City mainly came from large-scale commercial farms, while those from Zigong City were sourced from small-scale backyard farms, and those from Qingdao City were obtained from prominent zoological facilities. Additionally, 157 throat swab samples and 108 serum samples were collected from personnel working in these locations who may have direct contact with birds during their duties (Table 1).

**Table 1:** Number of samples and facilities collected from different cities.

city			Commercial farms	Backyard farms	Slaughterhouses	Zoos
Zigong, Sichuan	No. of facilities		17	6	1	0
	Bird facal samples		53	47	1	0
	Poultry-related staff	Throat swab samples	70	39	0	0
		Serum samples	55	39	0	0
Liaocheng, Shandong	No. of facilities		5	0	0	0
	Bird facal samples		393	0	0	0
	Poultry-related staff	Throat swab samples	30	0	0	0
		Serum samples	0	0	0	0
Qingdao, Shandong	No. of facilities		1	0	0	5
	Bird facal samples		31	0	0	753
	Poultry-related staff	Throat swab samples	0	0	0	18
		Serum samples	0	0	0	14

During the process of gathering avian specimens, samples were collected from all species within a facility. Emphasis was placed on the health status of the birds, ensuring they exhibited no signs of associated illnesses at the time of sample collection, and efforts were made to obtain fresh fecal samples during this process. In the case of staff sample collection, the guiding criterion was to obtain throat swabs and blood samples from individuals who have sustained and regular exposure to aerosols containing bird feces and feathers, particularly caretakers. Throat swabs and fecal samples were preserved in 3 mL of sucrose–phosphate–glutamate (SPG) solution; all samples used in the present study were transported cryogenically and stored at −80°C until further analysis.

During the sample collection process, relevant health data and basic information were obtained from the workers. Six months later, biological samples were obtained from workers who had initially tested positive in throat swabs, and their health status was documented once more.

### DNA extraction

Before DNA extraction, 1 mL of each sample in SPG was aspirated and centrifuged at 20,913 × g for 30 min at 4°C. Then, the supernatant was discarded leaving 200 μL of liquid for DNA extraction using the QIAamp DNA Kit (Qiagen, Germany) according to the manufacturer’s instructions. For fecal samples, 250 mg of each stool sample was used to extract DNA using the QIAamp Power Fecal Pro DNA Kit (Qiagen) according to the manufacturer’s instructions. The final eluted DNA solution (100 μL) was divided into aliquots and stored at −20°C until use. In the DNA extraction process, the concentration was measured by using UV absorption spectrophotometry.

### Real-time fluorescence quantitative PCR

Each DNA sample was screened by real-time fluorescence quantitative PCR targeting the *ompA* gene of *C*. *psittaci* according to a previously described method [15]. DNA extracted from strains isolated from patients infected with *C*. *psittaci* was used as a positive control [16], while DNA extracted from *C*. *psittaci*-negative samples was used as a negative control. In the qPCR assay, a cyclic threshold (Ct) value ≥40 was defined as negative.

### Genotyping

The VDII region of the *ompA* gene was amplified from the qPCR-positive samples [17]. The amplicons were sequenced by the Tianyi Huiyuan Biotechnology Company (Beijing, China), and the obtained sequences were compared with the known *ompA* sequences of *C*. *psittaci* (reference sequences) using BLAST analysis in GenBank (http://blast.ncbi.nlm.nih.gov/). The sequences reported in this paper have been deposited in GenBank, the accession numbers are provided in S1 Table (OR759370-OR759407, OR734296—OR734303).

To assess the phylogenetic relationship of the strains in the positive samples with different genotypes of *C*. *psittaci*, a phylogenetic tree was constructed on the basis of *C*. *psittaci ompA* gene. The obtained novel sequences and the reference sequences of *C*. *psittaci ompA* were aligned using the ClustalW in MEGA 11.0. Phylogenetic trees were then estimated using the MEGA 11.0 maximum composite likelihood model by the neighbor-joining method, with 1000 bootstrap replications [18].

### Serological assay

Serum-specific IgG antibodies against *C*. *psittaci* were detected in serum samples using commercial indirect fluorescent antibody (IFA) assay kits (Savyon Diagnostics Ltd., Ashdod, Israel) following the manufacturer’s instructions, at the cutoff of 1:64. In brief, a two-fold serial dilutions (from 1:32 to 1:1024) of the serum samples were prepared in 96-well polystyrene plate(s). A titer of 1:64 or higher was considered positive for *C*. *psittaci* infection.

### Statistical analysis

Statistical analysis was conducted to analyze variations in the positive rates of fecal samples among different avian species, as well as the positive rates in throat swabs and serum samples from staff members of various facilities. The chi-square test was used for this analysis, with *P* < 0.05 indicating statistical significance.

## Results

### Poultry samples

**Fig 1:**
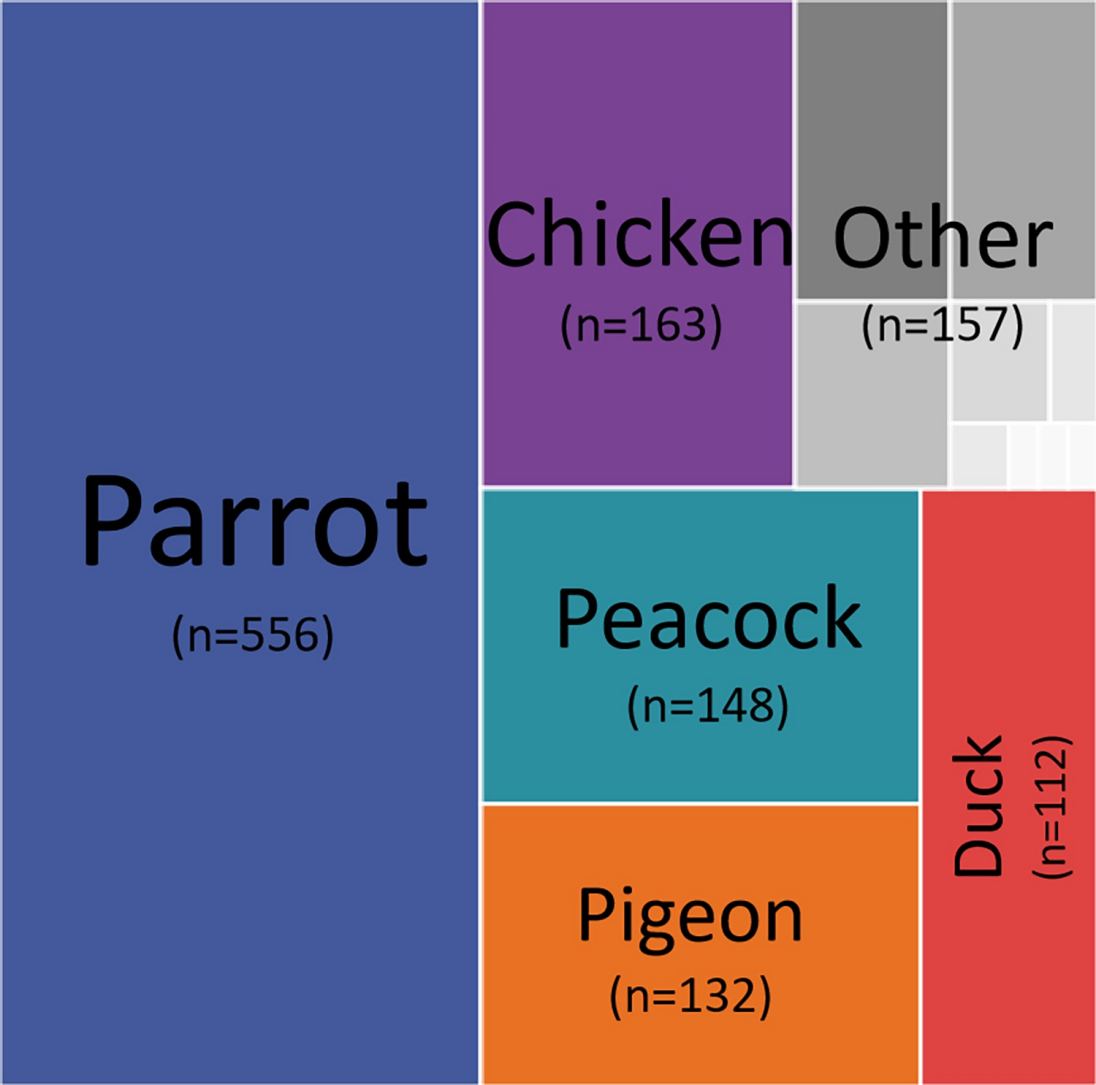
The number of different avian species.

A total of 1278 fecal samples of birds were collected from 35 different establishments in three cities in China for detection of *C*. *psittaci*. The fecal samples were collected from birds and poultries representing eleven avian species. The number of different avian species is presented in the Fig 1. Of the fecal samples, parrots was the most abundant (43.5%). A total of 40 samples (3.13%) were found to be positive for *C*. *psittaci ompA* by using qPCR. Specifically, positive samples were detected in peacock, parrot, duck, pigeon and chicken. The result are shown in Table 2. *C*. *psittaci* positive rates differed signifcantly (*P*<0.01) among samples collected from different avian species, with the samples from peacock having the highest rate (8.78%). The 40 positive samples, were from commercial farms and zoos, while no detection was reported in backyard farms and slaughterhouses. The detection rate in different facilities are presented in Table 2. The difference in detection rates between commercial farms and zoos was small, and statistical analysis showed no significant difference (*P>*0.05).

**Table 2:** Positive rate of fecal samples in different avian species and facilities.

Facal samples	Avian species						Facilities		
	Peacock	Parrot	Duck	Pigeon	Chicken	P	Commercial farms	Zoos	P
Positive rate	8.78% (13/148)	3.24% (18/556)	4.91% (6/122)	0.75% (1/132)	1.23% (2/163)	0.003	3.35% (16/477)	3.19% (24/753)	0.651

### Related-worker samples

**Fig 2:**
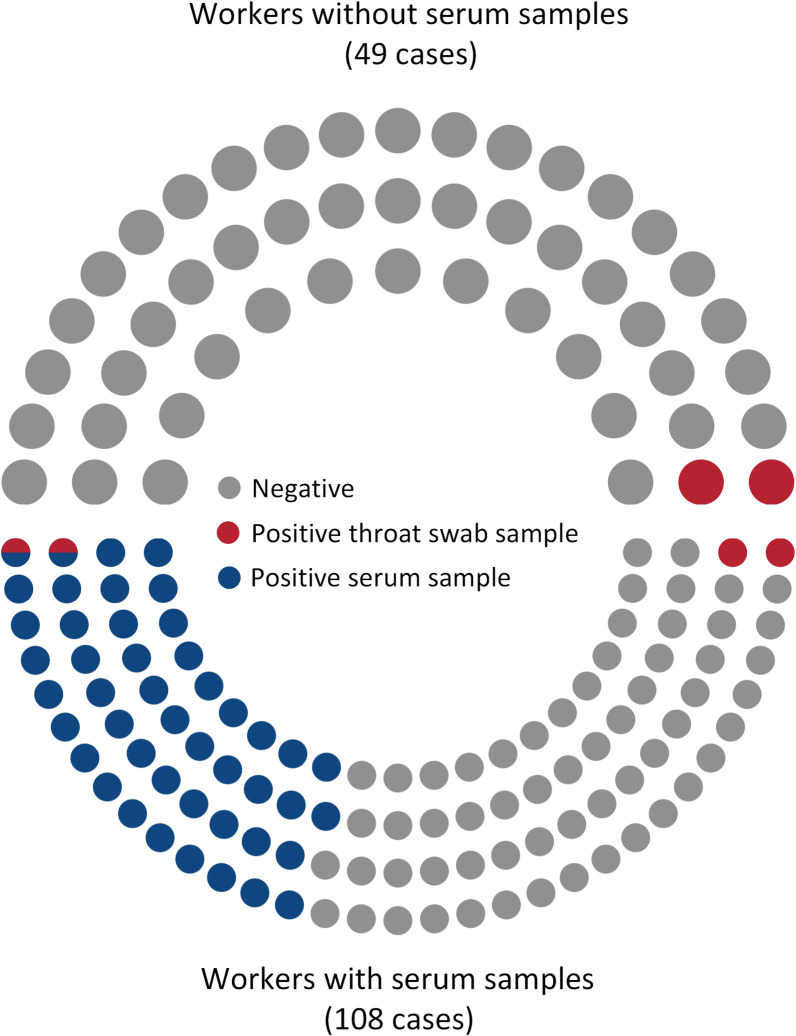
The detection result of workers samples.

In addition, we collected 157 samples of staff from these establishments. Among these, 108 staff members had both serum samples and throat swab samples collected, while 49 staff members only had throat swab samples collected. In the qPCR assay, only 3.82% (6/157) of the throat swab samples tested positive for *C*. *psittaci ompA*. Epidemiological investigations into the physical condition of staff members who tested positive found that worker infections were transient. In the IFA assay, 46 (42.59%) serum samples were positive for IgG against *C*. *psittaci*. Statistical analysis showed no significant difference in the detection rates of two assay among workers in various facilities. The results were presented in Table 3. Moreover, among the 46 staff members who had positive serum samples, two individuals were also tested positive for throat swab samples (Fig 2).

**Table 3:** Positive rate of related workers samples in different facilities.

Related worker samples	Throat swab samples			Serum samples			
	Commercial farms	Backyard farms	P	Commercial farms	Backyard farms	Zoos	P
Positive rate	2.00% (2/100)	10.26% (4/39)	0.05	34.55% (19/55)	53.85% (21/39)	42.86% (6/14)	0.176

### Genotyping

**Fig 3:**
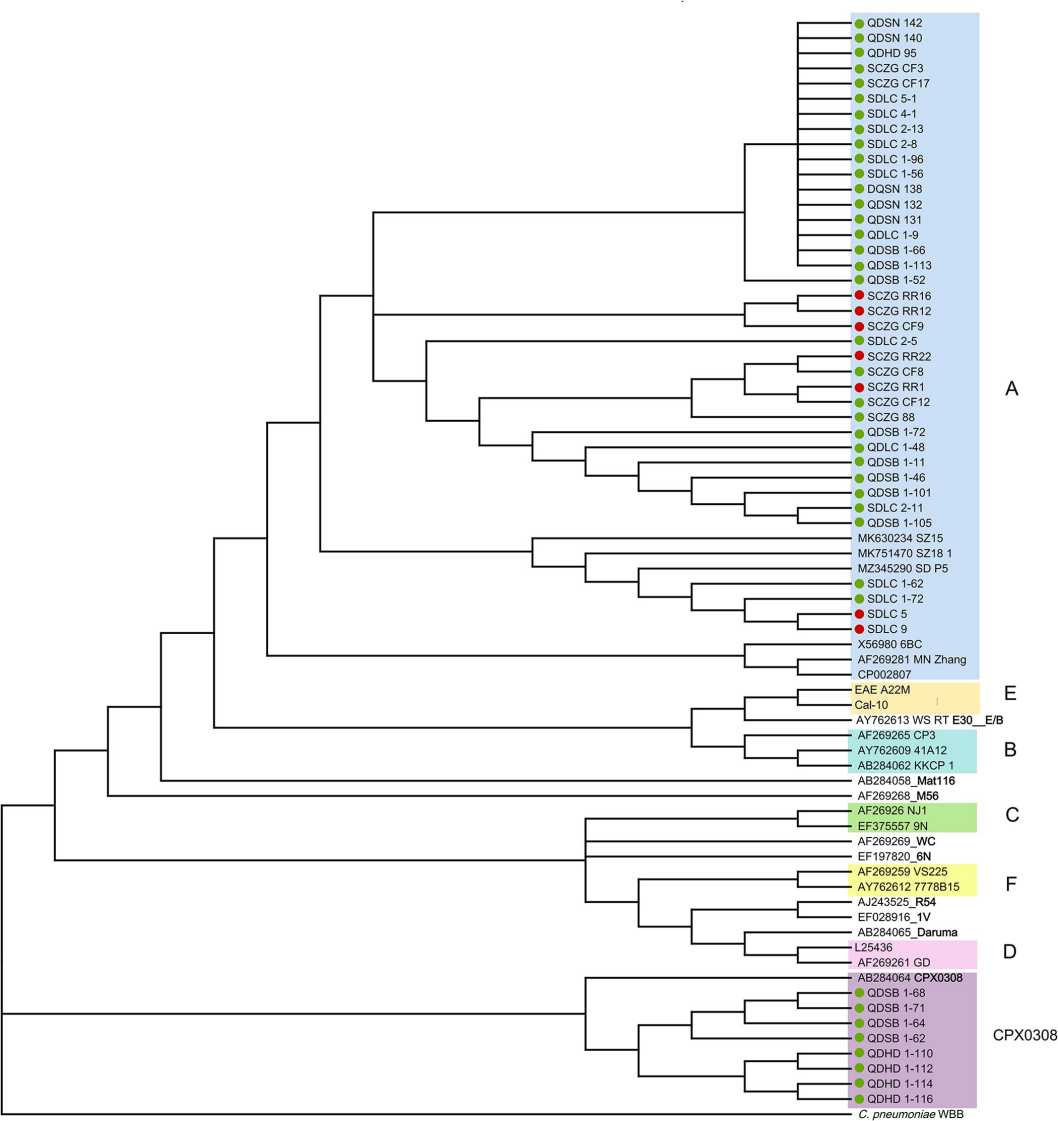
Phylogenetic analysis of the VDⅡ region sequence of the *ompA* gene of *C*. *psittaci*. The *ompA* gene sequences were compared and analyzed using MEGA 11.0 software. Phylogenetic trees were constructed by using the neighbor-joining method based on the general time-reversible model. Bootstrap tests were performed using 1000 repetitions. The samples with color markings in the figure were assessed in the present study: the red dot indicates employee samples, and the green dot indicates bird/poultry samples.

The strains detected in the samples were genotyped on the basis of their *ompA* sequences. The six strains from staff samples were all genotype A. Among the 40 strains detected in the bird and poultry samples, 32 strains were genotype A, and eight strains detected in peacock samples were genotype CPX0308. Of the 32 type A strains, five strains were detected in samples from peacock, eight strains from parrot, six strains from duck, two strains from chicken, and one strain from pigeon (Fig 3).

## Discussion

The present study investigated the prevalence of *C*. *psittaci* infection in birds and poultries and employees at farms and zoos in three cities in China. The study concluded that the positive rate in the poultry samples was 3.13%, which is similar to the incidence of 4% reported in a survey of symptomatic pet birds in southern Hunan, China [14]. A study of pigeons in the three eastern provinces of China showed a positive rate of 22.5% [19], which is higher than that found in this study. The significant difference in the positive rate between the two studies may be explained by use of dissimilar avian species and methods. The incidence in the present study is lower than that reported in South Korea (36.5%) [20] but similar to that reported in Sweden (2.2%) [21] and Poland (7.7%) [22]. These studies suggest that *C*. *psittaci* has a worldwide distribution and a wide range of natural hosts, especially birds and poultries.

Screening of these fecal samples by qPCR showed no statistical difference in the positive rates among sample groups from different facilities. The concentrated breeding in commercial farms leads to high animal density, resulting in increased production of feces and waste. Therefore, the likelihood of the spread of psittacosis caused by *C*. *psittaci* carried in aerosols containing pathogens and dust generated during cleaning will increase [23]. Furthermore, *C*. *psittaci* was not detected in the backyard farm during this test. This may be due to the smaller scale of backyard farming in China compared to other countries, as well as the limited sample size collected in the backyard farm during this study. However, the results showed that peacocks had the highest prevalence rate of *C*. *psittaci* infection, followed by parrots. These birds were located in zoos, which indicates a potentially significant risk of transmission of *C*. *psittaci* to zoo visitors. Therefore, appropriate precautions should be taken to prevent the transmission of *C*. *psittaci* from these birds to humans.

Additionally, 157 throat swab samples from employees were assessed using qPCR; six samples were found to be positive, indicating that the corresponding individuals were infected with *C*. *psittaci*. Subsequently, the epidemiological investigation revealed that they showed mild clinical symptoms during the sampling period. They were sampled again half a year later and tested negative for *C*. *psittaci*, indicating that they had a transient infection with *C*. *psittaci*. The serological test results indicated that a large proportion of employees at the farms or zoos may have previously been infected with *C*. *psittaci*. A research investigation conducted in Beijing, China, uncovered a significant prevalence of *C*. *psittaci* among both avian species (26.7%) and their human caregivers (23.9%) [24]. These results revealed that *C*. *psittaci* is an important zoonotic respiratory pathogen originating in birds; therefore, pathogen surveillance of *C*. *psittaci* on farms and zoos should be improved to prevent the transmission of *C*. *psittaci* among high-risk groups.

In the genotyping analyses, it was found that the *C*. *psittaci* strains detected in the bird fecal samples were mainly type A. In the present study, the genotype CPX0308 of *C*. *psittaci*, which was initially identified in an asymptomatic oriental white ibis in Japan, was detected in samples from peacocks [17]. The genotype has been reported in China for the first time. Notably, all the CPX0308-type strains were associated with peacocks, suggesting that strains with this genotype may have a host preference. Different genotypes exhibit host preferences (specific bird species), which can aid in determining transmission routes and hosts through genotyping positive samples [25]. In our study, it was found that the amplified genetic sequences of *C*. *psittaci* identified from employee samples had high homology with those identified from bird/poultry samples collected from the corresponding farms/zoos. Therefore, it is reasonable to believe that the *C*. *psittaci* strains detected in the employee samples were transmitted to humans from birds or poultries carrying the pathogen during their close contact. Therefore, the relevant occupational protection training of high-risk groups needs to be reinforced to prevent an outbreak of *C*. *psittaci* infection in the population.

The results of this study indicate a higher frequency of positive throat swab samples among individuals working in backyard farms than other facilities, suggesting a potential prevalence of acute infections and a risk of transmission between human individuals. Previous studies have also shown the plausibility of human-to-human transmission of *C*. *psittaci* [26]. Furthermore, the prevalence of positive serum samples on commercial farms was significantly higher than other facilities, indicating a higher incidence of *C*. *psittaci* infections among personnel in these settings. This finding suggests an increased susceptibility to infection among individuals working on commercial farms. Although the prevalence of acute and past infections among zoo staff was relatively low, the number of visitors to zoos is significant, and they may be exposed to airborne pathogens. Therefore, regular monitoring of bird-inhabited areas is crucial to minimize the potential for disease outbreaks.

## Conclusions

In this research, specimens were obtained from avian species and individuals associated with them for the purpose of identifying *C*. *psittaci*. This initiative aimed to determine the prevalence and geographical distribution of *C*. *psittaci* in China, while also gaining insights into the predominant genotypes. Research results show that *C*. *psittaci* is spreading among birds and workers. Therefore, enhancing the monitoring of *C*. *psittaci* in birds and implementing effective prevention and control measures, such as maintaining clean hygiene conditions, providing occupational training, and offering protective measures for workers, can help reduce the spread of pathogens in the population.

## Supporting information

S1 Table Basic information of the positive samples. (DOCX)
