# Supplementary material for: Prevalence and genotypes of Chlamydia psittaci in birds and related workers in three cities of China
Source: PLoS One. 2024 Aug 8;19(8):e0308532. doi: 10.1371/journal.pone.0308532 (PMC11309507; doi:10.1371/journal.pone.0308532)
Supplement: S1 Table — (DOCX) [file pone.0308532.s001.docx]

**Supplementary Table 1.**  Basic information of the positive samples.

|  | Case No. | Specie | City | Sample | Genotype | GenBank numbers | Time | Facility |
| --- | --- | --- | --- | --- | --- | --- | --- | --- |
| 1 | SDLC 5 | Staff | Liaocheng, Shandong | Throat swab | A | OR759380 | 2022 | Commercial farm |
| 2 | SDLC 9 | Staff | Liaocheng, Shandong | Throat swab | A | OR759381 | 2022 | Commercial farm |
| 3 | ZG RR22 | Staff | Zigong，Sichuan | Throat swab | A | OR759383 | 2023 | Backyard farm |
| 4 | ZG RR16 | Staff | Zigong，Sichuan | Throat swab | A | OR759384 | 2023 | Backyard farm |
| 5 | ZG RR12 | Staff | Zigong，Sichuan | Throat swab | A | OR759385 | 2023 | Backyard farm |
| 6 | ZG RR1 | Staff | Zigong，Sichuan | Throat swab | A | OR759386 | 2023 | Backyard farm |
| 7 | SDLC 1-56 | Parrot | Liaocheng, Shandong | Feces | A | OR759370 | 2022 | Commercial farm |
| 8 | SDLC 1-62 | Parrot | Liaocheng, Shandong | Feces | A | OR759371 | 2022 | Commercial farm |
| 9 | SDLC 1-72 | Parrot | Liaocheng, Shandong | Feces | A | OR759372 | 2022 | Commercial farm |
| 10 | SDLC 1-96 | Parrot | Liaocheng, Shandong | Feces | A | OR759373 | 2022 | Commercial farm |
| 11 | SDLC 2-5 | Parrot | Liaocheng, Shandong | Feces | A | OR759374 | 2022 | Commercial farm |
| 12 | SDLC 2-8 | Parrot | Liaocheng, Shandong | Feces | A | OR759375 | 2022 | Commercial farm |
| 13 | SDLC 2-11 | Parrot | Liaocheng, Shandong | Feces | A | OR759376 | 2022 | Commercial farm |
| 14 | SDLC 2-13 | Parrot | Liaocheng, Shandong | Feces | A | OR759377 | 2022 | Commercial farm |
| 15 | SDL C4-1 | Duck | Liaocheng, Shandong | Feces | A | OR759378 | 2022 | Commercial farm |
| 16 | SDLC5-1 | Parrot | Liaocheng, Shandong | Feces | A | OR759379 | 2022 | Commercial farm |
| 17 | ZG22 88 | Chicken | Zigong，Sichuan | Feces | A | OR759382 | 2022 | Commercial farm |
| 18 | ZG CF17 | Duck | Zigong，Sichuan | Feces | A | OR759387 | 2023 | Commercial farm |
| 19 | ZG CF12 | Duck | Zigong，Sichuan | Feces | A | OR759388 | 2023 | Commercial farm |
| 20 | ZG CF9 | Duck | Zigong，Sichuan | Feces | A | OR759389 | 2023 | Commercial farm |
| 21 | ZG CF3 | Duck | Zigong，Sichuan | Feces | A | OR759390 | 2023 | Commercial farm |
| 22 | ZG CF8 | Duck | Zigong，Sichuan | Feces | A | OR759391 | 2023 | Commercial farm |
| 23 | QDHD 95 | Pigeon | Qingdao，Shandong | Feces | A | OR759392 | 2023 | Zoo |
| 24 | QDSN 140 | Parrot | Qingdao，Shandong | Feces | A | OR759393 | 2023 | Zoo |
| 25 | QDSN 142 | Parrot | Qingdao，Shandong | Feces | A | OR759394 | 2023 | Zoo |
| 26 | QDSN 138 | Parrot | Qingdao，Shandong | Feces | A | OR759395 | 2023 | Zoo |
| 27 | QDSN 132 | Parrot | Qingdao，Shandong | Feces | A | OR759396 | 2023 | Zoo |
| 28 | QDSN 131 | Parrot | Qingdao，Shandong | Feces | A | OR759397 | 2023 | Zoo |
| 29 | QDLC1-9 | Parrot | Qingdao，Shandong | Feces | A | OR759398 | 2023 | Zoo |
| 30 | QDLC1-48 | Parrot | Qingdao，Shandong | Feces | A | OR759399 | 2022 | Zoo |
| 31 | QDSB1-11 | Parrot | Qingdao，Shandong | Feces | A | OR759400 | 2022 | Zoo |
| 32 | QDSB1-46 | Parrot | Qingdao，Shandong | Feces | A | OR759401 | 2022 | Zoo |
| 33 | QDSB1-52 | Parrot | Qingdao，Shandong | Feces | A | OR759402 | 2022 | Zoo |
| 34 | QDSB1-66 | Peacock | Qingdao，Shandong | Feces | A | OR759403 | 2022 | Zoo |
| 35 | QDSB1-72 | Peacock | Qingdao，Shandong | Feces | A | OR759404 | 2022 | Zoo |
| 36 | QDSB1-101 | Peacock | Qingdao，Shandong | Feces | A | OR759405 | 2022 | Zoo |
| 37 | QDSB1-105 | Peacock | Qingdao，Shandong | Feces | A | OR759406 | 2022 | Zoo |
| 38 | QDSB1-113 | Peacock | Qingdao，Shandong | Feces | A | OR759407 | 2022 | Zoo |
| 39 | QDSB1-62 | Peacock | Qingdao，Shandong | Feces | CPX0308 | OR734296 | 2022 | Zoo |
| 40 | QDSB1-64 | Peacock | Qingdao，Shandong | Feces | CPX0308 | OR734297 | 2022 | Zoo |
| 41 | QDSB1-68 | Peacock | Qingdao，Shandong | Feces | CPX0308 | OR734298 | 2022 | Zoo |
| 42 | QDSB1-71 | Peacock | Qingdao，Shandong | Feces | CPX0308 | OR734299 | 2022 | Zoo |
| 43 | QDHD1-110 | Peacock | Qingdao，Shandong | Feces | CPX0308 | OR734300 | 2022 | Zoo |
| 44 | QDHD1-112 | Peacock | Qingdao，Shandong | Feces | CPX0308 | OR734301 | 2022 | Zoo |
| 45 | QDHD1-114 | Peacock | Qingdao，Shandong | Feces | CPX0308 | OR734302 | 2022 | Zoo |
| 46 | QDHD1-116 | Peacock | Qingdao，Shandong | Feces | CPX0308 | OR734303 | 2022 | Zoo |
